# Private Equity Acquisitions of Assisted Living

**DOI:** 10.1093/geroni/igaf122.1403

**Published:** 2025-12-31

**Authors:** Kali Thomas, Momotazur Rahman, Sheryl Zimmerman

## Abstract

Private equity (PE) is a growing modality of investment in healthcare and affecting various populations, including older adults. However, within the long-term care sector, previous studies only focus on PE’s impact on nursing homes. To meet the unmet gap in the literature, this panel assembles five presenters who will discuss their research to identify and understand PE’s activities in assisted living (ALs) - the largest residential provider of long-term care in the US. The first presenter will share findings from a systematic review describing the measurement of PE ownership of healthcare and associated potential measurement issues. In order to identify PE ownership in ALs, the second presenter will share a novel approach to tease out complex corporate structures of ALs -- an essential step to measure PE’s ownership. Because PE acquisitions mostly focus on chain-affiliated providers and yet AL chain information is not available, the third presenter identifies and compares the corporate chain ownership of AL communities. Using the innovations described by the second and third presenters, the fourth panelist shares findings from identifying PE ownership in ALs and outlines the growing trends over the past decade. The fifth presenter provides valuable, qualitative information from extensive in-depth semi-structured interviews with PE and AL stakeholders to provide a comprehensive understanding of PE involvement in AL. The discussant is the director of the Center for Excellence in Assisted Living at UNC and will contextualize these panelists’ findings in the current research and regulatory landscape and discuss implications for resident care. This is a collaborative symposium between the Assisted Living and Research in Quality of Care Interest Groups. Assisted Living Interest Group Sponsored Symposium

